# A clinical pathway for pre-operative screening of COVID-19 and its influence on clinical outcome in patients with traumatic fractures

**DOI:** 10.1007/s00264-020-04645-3

**Published:** 2020-05-28

**Authors:** Yutong Meng, Kunpeng Leng, Lei Shan, Meng Guo, Junlin Zhou, Qingxian Tian, Yong Hai

**Affiliations:** Department of Orthopaedics, Beijing Chaoyang Hospital, Capital Medical University, Beijing, China

**Keywords:** Traumatic fracture, COVID-19 screening, Delayed surgery, Perioperative complication, Clinical safety

## Abstract

**Purpose:**

Coronavirus disease 2019 (COVID-19) has become a worldwide pandemic. The toughest issue traumatic orthopaedic surgeons are faced with is how to maintain a balance between adequate COVID-19 screening and timely surgery. In this study, we described our experience with pre-operative COVID-19 screening in patients with traumatic fractures. Furthermore, we analysed the clinical results of fracture patients undergoing confined or emergency surgery during the COVID-19 outbreak.

**Methods:**

This was a case series study. Patients with traumatic fractures who were admitted to our hospital for surgery were enrolled in this study during the COVID-19 outbreak from March to April 2020. All patients were enrolled and managed using the standardized clinical pathway we designed for preoperative COVID-19 screening. Clinical, laboratory and outcome data were analysed.

**Results:**

The average surgery waiting time from injury to surgery was 8.7 ± 3.4 days. The average waiting time from admission to surgery was 5.3 ± 2.8 days. These average waiting times were increased by 4.1 days and 2.0 days, respectively, compared with 2019 data. Cardiovascular complications, venous thromboembolism and pneumonia occurred in one, two and one patient, respectively. Three and two patients developed pre-operative and postoperative fevers, respectively.

**Conclusions:**

We introduced a novel clinical pathway for pre-operatively screening of COVID-19 in traumatic orthopaedic patients. The delay in surgery caused by COVID-19 screening was minimized to a point at which reasonable and acceptable clinical outcomes were achieved. Doctors should pay more attention to perioperative complications, such as cardiovascular complications, venous thromboembolism, pneumonia and fever.

## Introduction

The World Health Organization declared coronavirus disease 2019 (COVID-19) a pandemic on March 11, 2020 [1]. COVID-19 has spread to 3,064,823 people, resulting in 211,607 deaths worldwide as of April 28, 2020. On March 14, 2020, the US Surgeon General suggested stopping all elective surgeries. However, the toughest problem traumatic orthopaedic surgeons faced is not elective surgery but emergency or confined to surgery on patients with traumatic fractures.

Screening for COVID-19 is very important. Rashly performed surgery without excluding COVID-19 increases the risk of COVID-19 contamination in the hospital, which exposes patients and doctors to grave danger [2]. However, COVID-19 screening is time-consuming, which delays surgery and may result in malunion, disability of an extremity, or a more serious complication [3]. Thus, maintaining a balance between adequate screening and timely surgery is essential for peri-operative management of traumatic fractures.

Little was known about managing emergency or confined to surgery for traumatic fractures during the initial phase of the COVID-19 global crisis. In this study, we describe our experience with pre-operative screening of COVID-19 in patients with traumatic fractures to maintain the safety of medical activities. Furthermore, we analysed the clinical manifestations and results of fracture patients undergoing confined or emergency surgery during the COVID-19 outbreak. This study provides orthopaedic surgeons with valuable information on peri-operative management of traumatic fracture patients and will ultimately help to manage the COVID-19 epidemic.

## Materials and methods

### Study design

This was a case series study. We designed a standardized clinical pathway to pre-operatively assess COVID-19 in traumatic fracture patients. All patients in this study were managed strictly and enrolled in this clinical pathway. Subsequently, timely surgery was performed on all patients.

### Inclusion and exclusion criteria

Patients with traumatic fractures who were admitted to our hospital were enrolled in this study during the COVID-19 outbreak from March to April 2020. Patients were excluded if their age was less than 18 years, their disease course was > three weeks, or they chose conservative treatment.

### Data collection and statistics

Epidemiological, demographic, clinical, laboratory, treatment and outcome data were extracted from electronic medical records using a standardized data collection form and subsequently analysed. The following variables were extracted: delayed admission, delayed operation, cardiovascular complications, venous thromboembolism, pneumonia, incision complications and peri-operative fever. Continuous variables were expressed as mean ± standard deviation (SD). The categorical data were expressed as number and percentage (%).

### Definitions

According to the latest version (7th) of the Diagnosis and Treatment Protocol for COVID-19 released by the China National Health Commission on March 3, 2020 [4], a suspect case is defined based on the patient’s epidemiological history and clinical manifestations. A history of travel to or residence in a highly epidemic area or its surrounding areas is one of the epidemiological constituent conditions. Contact with COVID-19-infected patients, presence of a fever, or contact with patients with respiratory symptoms from a highly epidemic area and its surrounding areas within 14 days are also epidemiological constituent conditions. Clinical manifestations, such as fever and/or respiratory symptoms, specific computed tomography (CT) imaging characteristics and specific abnormalities in white blood cell or lymphocyte counts during the early stage of onset were important factors. A suspect case was considered if there was an epidemic history plus any two of the above clinical manifestations or all of the above clinical manifestations if there was no clear epidemic history. A confirmed case was defined as a positive result by real-time reverse-transcription polymerase chain reaction in respiratory specimens or specific IgM and IgG detection in serum.

Inpatient wards were classified as isolation, buffer and general wards based on the level of protection. The isolation ward was mainly for patients with a high suspicion of COVID-19 infection. The buffer ward was mainly for patients who had little possibility of infection, but the possibility of COVID-19 infection could not be excluded, thus requiring observation and further examination. The doctors in the isolation and buffer wards, which are located in an isolated building, were equipped with the strictest personal protective equipment (PPE), such as N95 respirators, protective eyewear and protective coveralls. Each room in the isolation and buffer wards was restricted to one patient. Doctors in the general ward were equipped with basic PPE. All patients wore face masks. Family and friend visits were forbidden during hospitalization. Each patient was cared for by professional care staff.

## Results

### Clinical pathway for pre-operative assessments of COVID-19

**Fig. 1:**
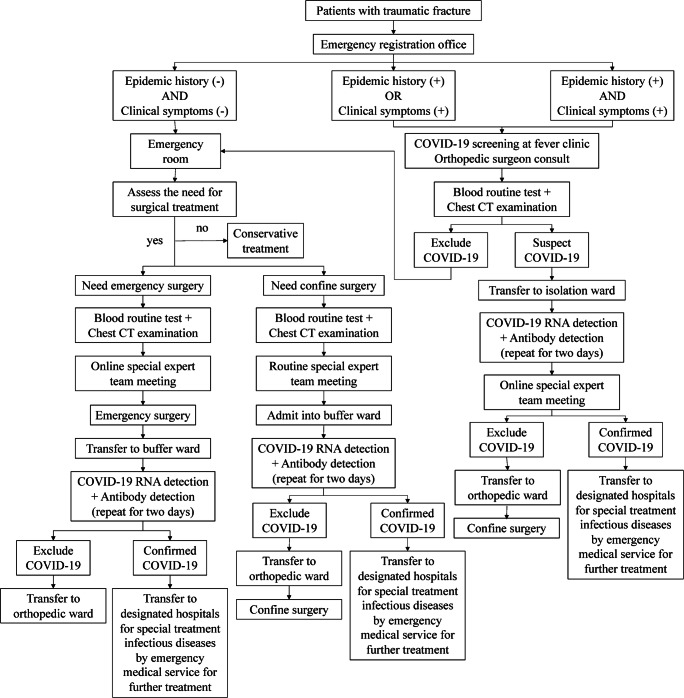
A clinical pathway on pre-operative screening of COVID-19 in patients with traumatic fractures

Since the COVID-19 outbreak began, our hospital designed a standardized clinical pathway to pre-operatively assess COVID-19 in February 2020, at the beginning of the COVID-19 outbreak in Beijing, China. The orthopaedics department has modified this clinical pathway according to the characteristics of the trauma patients. A detailed clinical pathway is shown in Fig. 1.

When patients first presented to the emergency room with a traumatic fracture, a brief screening for COVID-19 was performed immediately at the registration office. If they had no epidemiological history or clinical manifestations, they were allowed to enter the emergency room for further assessment. If the patient required emergency surgery, a routine blood test and chest CT examination were performed first. Then, as soon as all laboratory test results were ready, all medical records were uploaded and discussed at an online expert team meeting with five authoritative senior experts. Emergency surgery was performed if the COVID-19 diagnosis was not supported. After the surgery, the patient was transferred to the buffer ward for further treatment. If the patient needed confined surgery, all medical records were uploaded and discussed at a routine expert team meeting, which was held twice daily at 10 A.M. and 4 P.M. If the COVID-19 diagnosis was not supported, the patient was admitted to the buffer ward for further examination.

If the patient had an epidemic history or clinical symptoms, they were transferred to the fever clinic for further screening. If the blood test and pulmonary imaging features did not support a COVID-19 diagnosis, the patient was allowed back in the emergency room for further treatment. If the blood test and CT scans highly supported a COVID-19 diagnosis, the patient was transferred to the isolation ward immediately. Thereafter, further RNA and antibody detection assays were performed.

If a diagnosis of COVID-19 was confirmed at any time, related medical activities were suspended in the patient. The patient was transferred immediately by the emergency medical service to a designated hospital for special infectious disease treatment.

### Participant characteristics

During the study period, 43 patients were enrolled in this study. Of these patients, 29 with no epidemic history or clinical symptoms were categorized as relatively safe and allowed to enter the emergency room. Twelve patients had an epidemic history, and two patients had a cough or mild fever. They were transferred to the fever clinic immediately. After careful assessment, they were allowed to return to the emergency room for further treatment. Of these patients, 41 received confined surgery, and two received emergency surgery.

**Fig. 2:**
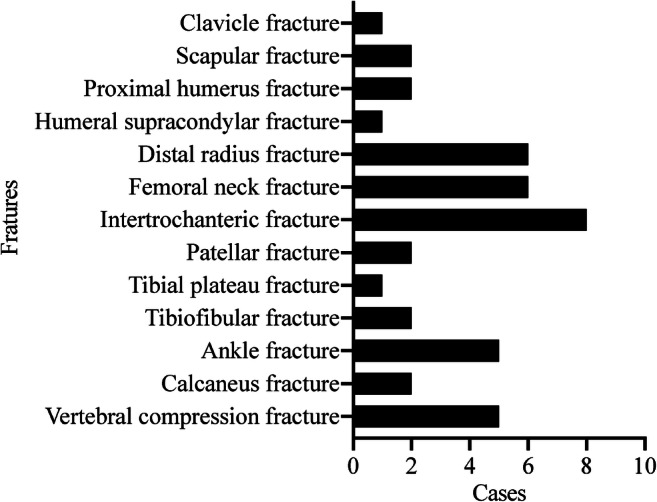
Distribution of the number of patients with fractures at different sites

**Fig. 3:**
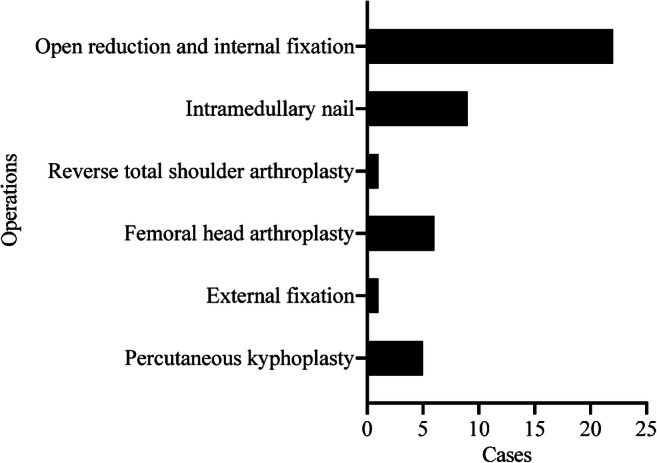
Distribution of the number of patients who received different surgical methods

The distribution of the fracture sites is listed in Fig. 2. Eight, six and six patients suffered from intertrochanteric, femoral neck and distal radius fracture respectively. Other fracture sites were evenly distributed. The distribution of surgery types is listed in Fig. 3. Twenty-two patients received open reduction and plate fixation, nine patients received intramedullary nail fixation, one patient received a reverse total shoulder arthroplasty, six patients received a femoral head arthroplasty, one patient received temporary external fixation and five patients received percutaneous kyphoplasty.

### Surgery waiting time

The average surgery waiting time from injury to surgery was 8.7 ± 3.4 days. The average waiting time from admission to surgery was 5.3 ± 2.8 days. The historical average waiting time from injury to surgery during March–April 2019 was 4.6 ± 2.6 days. The average waiting time from admission to surgery was 3.3 ± 1.7 days. The average waiting times were increased by 4.1 days and 2.0 days compared with 2019 data, respectively.

### Peri-operative complications

**Fig. 4:**
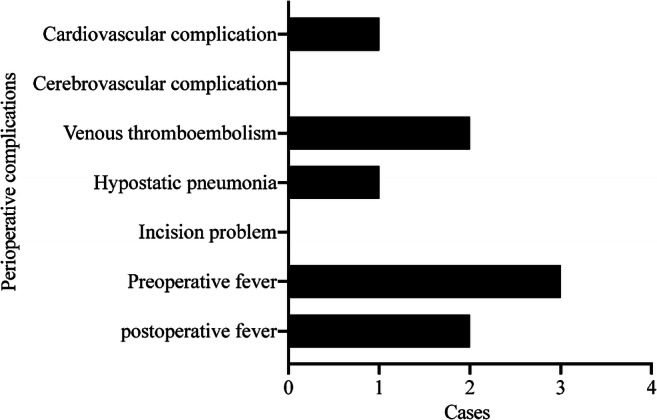
Distribution of the number of patients who developed a complication since the injury

The peri-operative complications are shown in Fig. 4. Peri-operative complications occurred in three patients. One 77-year-old male patient with a femoral neck fracture received femoral head arthroplasty on day four after admission. We detected increased serum levels of myocardial enzymes after surgery. After consultation with the cardiology department, this patient was diagnosed with mild cardiac failure and is currently under treatment in the surgical intensive care unit in stable condition. Another 84-year-old female patient with a femoral neck fracture received femoral head arthroplasty on day six after admission. We detected a venous thrombus in a lower extremity before surgery. There was also a 90-year-old female patient with an intertrochanteric fracture who received intramedullary nailing on day four after admission. We detected a venous thrombus in a lower extremity and mild pneumonia before surgery.

Three patients had a pre-operative fever, and two had a postoperative fever. After consultation with the infectious disease department, the possibility of COVID-19 infection was ruled out. According to the laboratory and imaging results, we confirmed that the preoperative fever was caused by a urinary infection or pneumonia. The reason for post-operative fever was absorption fever.

## Discussion

COVID-19 has the characteristics of rapid transmission, highly infectious, insidious onset and long incubation period [5]. It causes a significant challenge for clinical activity and the safety of traumatic orthopaedic physicians. The general ward and operating rooms are semi-open spaces. The consequences of admitting patients who unknowingly carry the disease into the general ward may be extreme because the entire inpatient ward and surgical team could be directly exposed to the virus. Thus, we proposed that strict COVID-19 screening is essential for those patients who need emergency or confined surgery.

We proposed a clinical pathway for perioperative COVID-19 screening. This clinical pathway aimed to exclude COVID-19 cases during the beginning of the clinical activity to minimize the possibility of nosocomial transmission of the virus as much as possible. Additionally, this clinical pathway contained emergency handling methods in cases of emergency. However, some other studies proposed different protocols. Zhen et al. proposed that patients requiring urgent or early orthopaedic care should present at the hospital as soon as possible with no difference from ordinary workflow [6]. Mohamed et al. conducted a study on operating room protocols for urgent surgery in orthopaedic patients with suspected or confirmed COVID-19 [7]. Ricardo et al. also proposed a setting and workflow within the operating room for COVID-19-positive trauma patients [8]. Most of these studies focused on the operating room setting and the PPE of medical workers, rather than the screening pathway.

Pre-operative screening for COVID-19 will inevitably increase the surgery waiting time. In this study, patients waited an average of 5.3 days before surgery, which was 2.0 days longer than the historical waiting period. Interestingly, the time from injury to surgery was 4.1 days longer than the historical time. Thus, the patients waited 2.1 days longer at home compared with before the outbreak. Since the beginning of the COVID-19 outbreak, the local government has encouraged all citizens to reduce unnecessary outdoor activities [5]. Most trauma patients were afraid to go outside or did not realize the severity of their traumatic condition. They chose to stay at home for temporary observation, thereby increasing the time from injury to admission. In addition, the 2-day COVID-19 RNA and antibody detection assays were indispensable when most patients were allowed to enter the buffer wards. This could explain the delay from admission to surgery.

There is conflicting evidence on the relationship between the delay to surgery and clinical outcomes in traumatic fracture patients. Rai et al. reported no difference in survival or functional outcomes between early surgery and late surgery [9]. A similar result was reported by Magaziner et al. [10]. However, Orosz et al. determined that clinical factors delayed surgery in trauma patients [11]. Yusuke et al. found that early surgery is associated with lower risks of pneumonia and pressure ulcers. However, early surgery was not associated with 30-day mortality or pulmonary embolism rates [12]. Daniel et al. revealed that an increased waiting time was associated with higher risks of clinical complications and 30-day mortality in hip fracture patients undergoing surgery [13]. In our study, the complication rate was 3/43 (7.0%). We believe that the clinical complications caused by the delay were reasonable and acceptable.

The most serious complication in this study was venous thromboembolism. Two elderly patients with hip fractures suffered from venous thromboembolism in a lower extremity. These two patients stayed at home for more than ten days without any anticoagulation before arriving at the emergency room. Of the patients who do not receive any venous thromboembolism prophylaxis, 40–60% undergoing hip surgery develop venous thromboembolism [14]. To avoid associated risks, the ACCP guidelines encourage using anticoagulation therapy for at least ten to 14 days and up to 35 days [15]. Ktistakis et al. proposed that low-molecular-weight heparin should be administered on admission to hip fracture patients, i.e., the sooner the better [16].

Another serious complication was pneumonia. Pneumonia is one of the most common and serious complications among elderly patients [17]. Joseph et al. revealed that pre-operative pneumonia is associated with a higher risk of adverse events and death after hip fracture surgery [18]. The risk factors for peri-operative pneumonia have been identified [19]. According to Seong-Eun et al., age, low body mass index, malnutrition, longer surgery duration and delayed surgery are risk factors for pneumonia [20]. Daniel et al. proposed that the risk factors for pneumonia in geriatric patients undergoing surgery for hip fracture are male sex, elderly age, low body mass index and chronic obstructive pulmonary disease [21]. In our study, a 90-year-old female patient with an intertrochanteric fracture who received intramedullary nailing on day four after admission had mild pneumonia before surgery. She stayed at home and remained in bed for 11 days before coming to the emergency room. Under such circumstances, the occurrence of pneumonia seemed inevitable.

Peri-operative fever during the COVID-19 epidemic is a difficult situation, as we cannot know for sure whether these patients have an ordinary infection or COVID-19. Thus, once we know a patient has a fever, the top priority is to suspend all clinical activity immediately and transfer the patient to a single room for isolation. Then, a careful examination is performed, including routine blood and urine tests, COVID-19 RNA and antibody detection assays and chest CT. The postoperative fever rate can be as high as 49.6% in hemiarthroplasty of elderly patients with femoral neck fractures. The main reasons for postoperative fever are pneumonia or a urinary tract infection [22]. Robert et al. revealed that the prevalence of post-operative fever in an orthopaedic trauma population is 18%. The main reasons for post-operative fever can be a urinary tract infection, pneumonia, or an incisional infection [23]. Bobin et al. analysed the characteristics and early prognosis of COVID-19 infection in fracture patients. The most common signs were fever, cough and fatigue at the time of presentation. Other than that, lymphopenia, high D-dimer level, high C-reactive protein level and characteristic CT findings, as well as RNA detection assays can help differentially diagnose COVID-19 from a common infection [24]. According to the laboratory and imaging results in this study, we confirmed that the reasons for pre-operative fever were urinary infection and pneumonia. The reason for post-operative fever was absorption fever.

### Limitations

Several limitations to our work should be discussed. Firstly, this study was a case series study, not a randomized controlled trial or cohort study. All patients were managed under the same proposed pathway. Setting up a control group was not feasible or ethical. Because a blank control would inevitably put all doctors and patients in grave danger. Thus, we chose to set up only one group. Secondly, as the number of patients was relatively small, selection bias was a concern. Citizens were encouraged to stay at home during the COVID-19 pandemic. Thus, the number of patients with fractures caused by outdoor activities was greatly reduced. We only enrolled 43 patients during the study period. The results of this study have reference value, so the relatively small scale of the study was not a problem. Our findings should be confirmed in a larger-scale randomized trial in the future. Finally, co-treatment bias should also be taken into account. To avoid potential co-treatment bias caused by different surgeons and additional treatments, all surgeries in this study were performed by the same surgical team, consisting of two chief physicians and three senior physicians. Except for the COVID-19 screenings, all other clinical work was performed as usual.

## Conclusions

In this study, we introduced a novel clinical pathway to pre-operatively screen for COVID-19. Based on this clinical pathway, we assured the safety of medical activities from interference by COVID-19. The delay of surgery caused by COVID-19 screening was minimized to a point at which reasonable and acceptable clinical outcomes were achieved. Our study also suggests that doctors should pay more attention to peri-operative complications, such as cardiovascular complications, venous thromboembolism, pneumonia and fever, under such a pre-operative screening pathway.

## Data Availability

Not applicable.
